# Trafficking regulates the subcellular distribution of voltage-gated sodium channels in primary sensory neurons

**DOI:** 10.1186/s12990-015-0065-7

**Published:** 2015-09-30

**Authors:** Lan Bao

**Affiliations:** State Key Laboratory of Cell Biology, Institute of Biochemistry and Cell Biology, Shanghai Institutes for Biological Sciences, Chinese Academy of Sciences, 320 Yue Yang Road, Shanghai, 200031 China

**Keywords:** Voltage-gated sodium channel, Primary sensory neuron, Trafficking regulation

## Abstract

Voltage-gated sodium channels (Na_v_s) comprise at least nine pore-forming α subunits. Of these, Na_v_1.6, Na_v_1.7, Na_v_1.8 and Na_v_1.9 are the most frequently studied in primary sensory neurons located in the dorsal root ganglion and are mainly localized to the cytoplasm. A large pool of intracellular Na_v_s raises the possibility that changes in Na_v_ trafficking could alter channel function. The molecular mediators of Na_v_ trafficking mainly consist of signals within the Na_v_s themselves, interacting proteins and extracellular factors. The surface expression of Na_v_s is achieved by escape from the endoplasmic reticulum and proteasome degradation, forward trafficking and plasma membrane anchoring, and it is also regulated by channel phosphorylation and ubiquitination in primary sensory neurons. Axonal transport and localization of Na_v_s in afferent fibers involves the motor protein KIF5B and scaffold proteins, including contactin and PDZ domain containing 2. Localization of Na_v_1.6 to the nodes of Ranvier in myelinated fibers of primary sensory neurons requires node formation and the submembrane cytoskeletal protein complex. These findings inform our understanding of the molecular and cellular mechanisms underlying Na_v_ trafficking in primary sensory neurons.

## Background

Voltage-gated sodium channels (Na_v_s) comprise the rising phase of action potentials and are therefore a critical factor in neuronal excitability. Na_v_s contain α and β subunits; however, α subunits alone execute channel functions. To date, nine isoforms of the α subunit (Na_v_1.1–1.9), which display various channel properties and selective tissue distribution, have been discovered. Na_v_1.7, Na_v_1.8 and Na_v_1.9 are peripheral Na_v_s that are highly and selectively expressed in primary sensory neurons located in the dorsal root ganglion (DRG). Recent progresses have revealed the importance of N_v_s in human pain disorders, especially Na_v_1.7, Na_v_1.8 and Na_v_1.9 [1, 2]. In general, the function of Na_v_s is regulated by their expression level, channel properties and subcellular distribution. Here, we focus on the regulation of the subcellular distribution of Na_v_s in adult primary sensory neurons by Na_v_ trafficking.

## Cellular and subcellular distribution of Na_v_s in primary sensory neurons

The cellular distribution of Na_v_s in primary sensory neurons is mainly detected by in situ hybridization and immunohistochemistry and, more recently, by single-cell polymerase chain reaction (PCR) and RNA sequencing. The electrophysiological detection of voltage-gated sodium currents in individual neurons also significantly helps to identify functional channel expression. In adult primary sensory neurons, three tetrodotoxin-sensitive (TTX-S; Na_v_1.1, Na_v_1.6 and Na_v_1.7) and two tetrodotoxin-resistant (TTX-R; Na_v_1.8 and Na_v_1.9) sodium channels have been identified. High level of TTX-S Na_v_1.2 and Na_v_1.3 is embryonically expressed in DRG neurons and dramatically decreased after post-natal, whereas Na_v_1.3 is re-expressed under certain pathological conditions that involve peripheral nerve injuries. DRG neurons are usually divided by size into small neuron (such as <800 µm^2^ in mice) and large neuron (>800 µm^2^) subsets that primarily consist of nociceptors and mechanoreceptors, respectively. Extensive recent studies using in situ hybridization have revealed that Na_v_1.1 and Na_v_1.6 are mainly expressed in 200 kDa neurofilament subunit (NF200)-positive neurons and that high levels of Na_v_1.7, Na_v_1.8 and Na_v_1.9 are found in NF200-negative neurons [3, 4]. High levels of Na_v_1.7 and Na_v_1.8 are also detected in NF200/tropomyosin receptor kinase A (TrkA)-positive neurons, and Na_v_1.7 is additionally expressed in half of the NF200-positive and TrkA-negative neurons [4]. Recent transcriptional profiling by single-cell PCR has confirmed that Na_v_1.7, Na_v_1.8 and Na_v_1.9 are enriched in both the isolectin B4 (IB4)-positive and the IB4-negative SNS-Cre/TdTomato populations, while Na_v_1.1 and Na_v_1.6 are mainly expressed in Parvalbumin-Cre/TdTomato neurons [5]. Each isoform may exhibit a different distribution pattern when detected by immunohistochemistry compared with in situ hybridization. Although antibodies are generally specific and sensitive, some antibodies only recognize specific protein structures in a subset of cells under particular conditions. In addition, regulation of protein translation may cause a divergence in protein and mRNA levels and lead to further discrepancies between the cellular distributions detected by the two methods.

Although combined methods are used to discover the cellular distribution pattern of Na_v_ channels, subcellular localization can only be detected by immunohistochemistry. In primary sensory neurons, Na_v_1.6, Na_v_1.7, Na_v_1.8 and Na_v_1.9 are the most frequently studied and display a primarily intracellular localization in the cell body, as detected by specific antibodies [6–11]. Na_v_1.6 and Na_v_1.7 are also localized to the nodes of Ranvier in myelinated fibers of the sciatic nerve and distributed throughout unmyelinated fibers of the sciatic nerve and dorsal root [7, 10, 12, 13]. Na_v_1.7 localizes to peripheral terminals in the skin and central terminals in the dorsal horn [7]. Na_v_1.8 is distributed in afferent fibers of the sciatic nerve and dorsal root [8, 11] and in axons of cultured DRG neurons [14] and further revealed to be localized at lipid rafts, especially in the axons of cultured small DRG neurons and sciatic nerve [15]. Na_v_1.9 preferentially localizes along axons of the IB4-positive unmyelinated fibers in the sciatic nerve [16].

In general, Na_v_s must be inserted into the plasma membrane of cell bodies and axons to function in neurons. A large pool of intracellular Na_v_s suggests the possibility that alterations in the mode of Na_v_ trafficking could lead to quick changes in channel and neuron function. In the physiological condition, an efficient expression of Na_v_s on the cell surface and in the axon of DRG neurons is in favor of primary sensation. However, an excessive increase of Na_v_ trafficking to cell surface in pathological conditions including peripheral inflammation and nerve injury induces abnormally neuronal excitability, which may reduce the response threshold of DRG neurons and involve in the development of pathological pain.

## Strategy to study the mechanisms that regulate Na_v_ trafficking

Na_v_s, similar to other membrane proteins, are synthesized in the rough endoplasmic reticulum (ER) and transported via vesicles. Regulation of the trafficking of these proteins can provide a quick and highly efficient way for cells to respond to the extracellular environment aside from transcriptional regulation. The molecular mechanisms that regulate Na_v_ trafficking depend primarily on three components: amino acids, motifs or sequences located within the channels that mediate the regulation, interacting proteins that respond to signals and connect with the trafficking machinery, and extracellular factors that transfer changes in the extracellular environment to neurons.

**Fig. 1:**
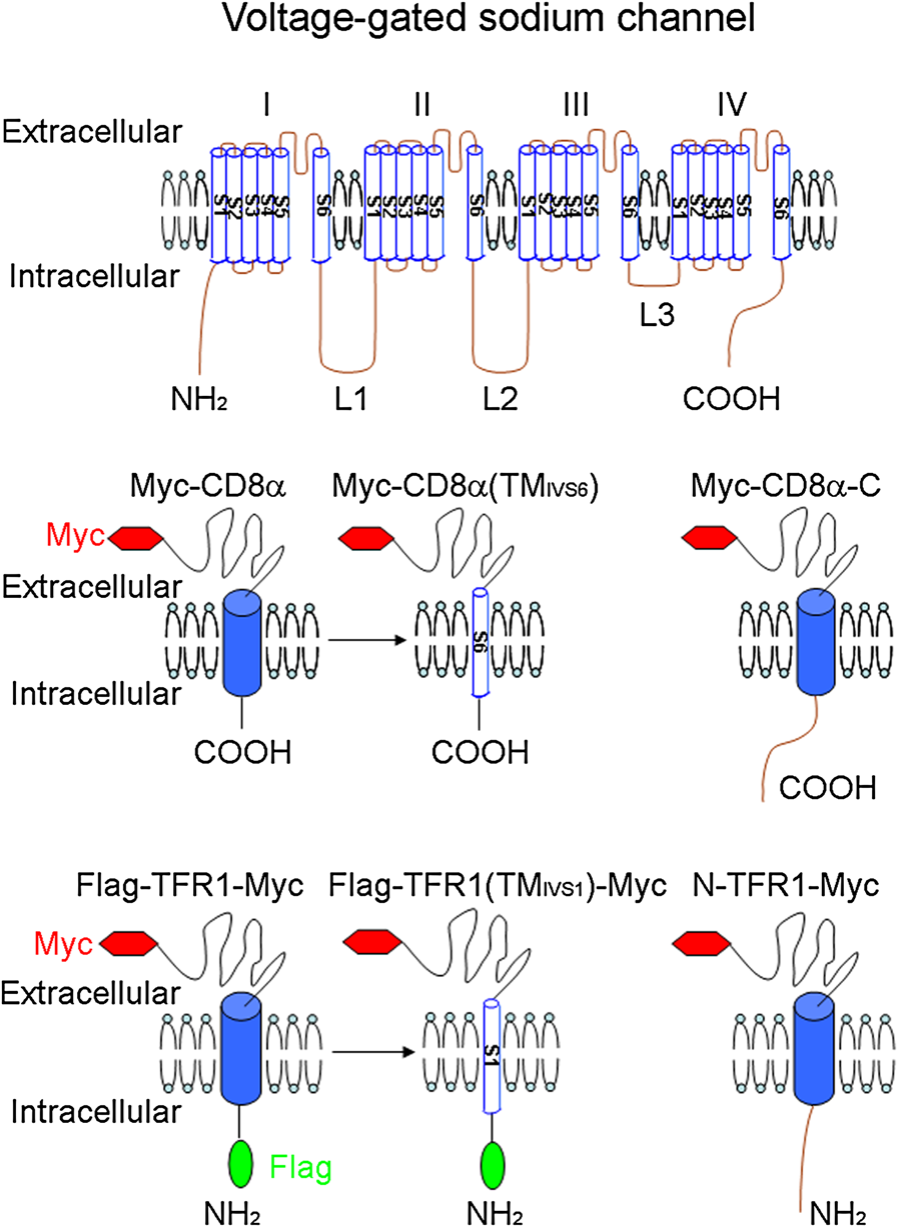
Model molecules to identify the signals in Na_v_s that mediate the trafficking regulation. Na_v_s consist of four domains (I, II, III and IV) connected by three intracellular loops (L1–L3); each domain is formed by six transmembrane segments (TM; S1–S6). Both the N-terminus (N) and the C-terminus (C) of Na_v_s are located in the cytoplasm. CD8α and TFR1, which have distinct cell-surface localization, are adapted to detect the roles that particular regions of the Na_v_s have in subcellular distribution. The type I membrane protein CD8α is suitable for testing three intracellular loops, the C-terminus and the transmembrane segments that pass through the membrane in the extracellular to intracellular direction (S2, S4 and S6), whereas the type II membrane protein TFR1 is appropriate for testing the N-terminus and the transmembrane segments that pass through the membrane in the opposite direction (S1, S3 and S5). A Myc tag is inserted to the N-terminus of CD8α or the C-terminus of TFR1, and non-permeabilized immunostaining is performed with Myc antibody in transfected living cells to label these proteins on the plasma membrane. A Flag tag is inserted to the N-terminus of TFR1 for the permeabilized immunostaining with Flag antibody to label the protein in whole cell. The sequence of CD8α or TFR1 is replaced with corresponding region of Na_v_, such as Myc-CD8α(TM_IVS6_), Myc-CD8α-C, Flag-TFR1(TM_IVS1_)-Myc and N-TFR1-Myc. This figure is adapted from Li et al. [15]

Na_v_s consist of four domains connected by three intracellular loops; each domain is formed by six transmembrane segments. Both the N-terminus and the C-terminus of Na_v_s are located in the cytoplasm. To identify the amino acids, motifs or sequences in Na_v_s that mediate the trafficking regulation, model molecules such as CD4, CD8α and transferring receptor 1 (TFR1), which have distinct cell surface localization, can be adapted to detect the roles that particular regions of the Na_v_s have in subcellular distribution (Fig. 1) [11, 17, 18]. To ascertain the effect of intracellular and transmembrane segments, the orientation of the Na_v_ intracellular sequence and transmembrane segment within the membrane should be considered when constructing the adapted molecule. The type I membrane protein CD8α is suitable for testing three intracellular loops, the C-terminus and the transmembrane segments that pass through the membrane in the extracellular to intracellular direction, whereas the type II membrane protein TFR1 is appropriate for testing the N-terminus and the transmembrane segments that pass through the membrane in the opposite direction (Fig. 1) [18]. Additionally, the specific cells used to examine distinct subcellular structures should be considered. For example, because neurons are round and their subcellular structures are occasionally obscured, COS-7 cells, which are derived from African green monkey kidney fibroblast-like cells and are flat, are usually used to analyze the subcellular localization of proteins in organelles. Importantly, the amino acids, motifs and sequences that are thought to mediate the regulation of Na_v_ trafficking should ultimately be tested by point mutation or sequence replacement in full-length channels to evaluate their role in subcellular localization.

Interacting proteins interact with specific amino acids, motifs or sequences within the Na_v_s to achieve the final trafficking of channels. Although a large-scale yeast two-hybrid screen for proteins that interact with the intracellular domain of Na_v_1.8 has been performed [19], more of the putative interacting proteins need to be confirmed, and proteins that interact with the extracellular and transmembrane domains of Na_v_s have yet to be discovered. A combination of mass spectrometry and immunoprecipitation with antibodies specific for Na_v_s may provide another approach for finding novel interacting proteins that mediate the regulation of Na_v_ trafficking. More importantly, the protein interaction could be regulated by extracellular factors that trigger signaling pathways to alter the protein activity. Large-scale screening for extracellular factors that regulate channel trafficking is limited by lack of the method for highly sensitive and efficient detection of the subcellular localization for membrane proteins.

## Surface expression of Na_v_s in primary sensory neurons

**Fig. 2:**
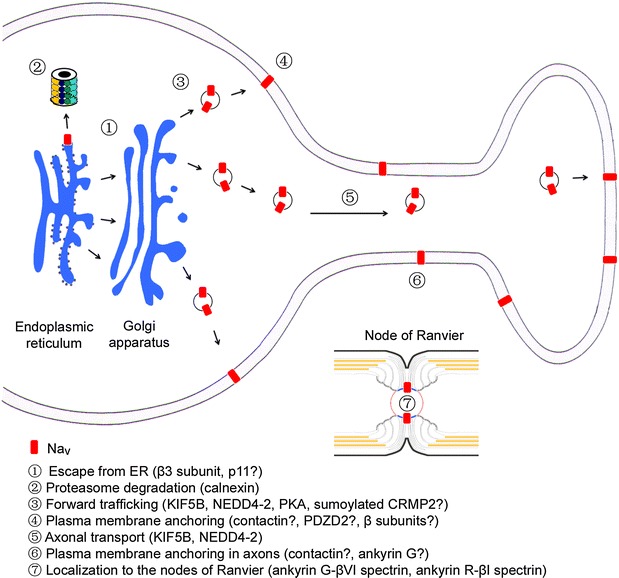
Main steps that regulates the subcellular distribution of Na_v_s in primary sensory neurons. The surface expression of Na_v_s is achieved by escape from the endoplasmic reticulum and proteasome degradation, forward trafficking and plasma membrane anchoring in primary sensory neurons. Axonal transport and localization of Na_v_s in afferent fibers involves motor proteins and scaffold proteins. Localization of Na_v_1.6 to the nodes of Ranvier in myelinated fibers of primary sensory neurons requires node formation and the submembrane cytoskeletal protein complex. The molecules listed are mostly positive regulators except NEDD4-2 that may impede forward trafficking of Na_v_1.7. However, the hypothesized roles of molecules with question mark during various steps of Na_v_ trafficking in primary sensory neuron need to be proved

High levels of Na_v_s are not localized on the plasma membrane of primary sensory neuron [6–11, 20]. The trafficking of the channels could be impeded at various points along the secretory pathway, including in the ER, Golgi complex and vesicles (Fig. 2). Na_v_1.8 displays a reticulum-like distribution and colocalizes with calnexin, an ER marker, in transfected COS-7 cells [11]. Using CD8α and TFR1 as model molecules to screen potential ER-localization motifs and sequences, an RXR motif in the first intracellular loop of Na_v_1.8 and several transmembrane segments containing acidic amino acids were found to be responsible for its ER localization [11, 18]. The β3 subunit interacts with Na_v_1.8 and masks the RXR motif to promote surface expression of the channel [11]. Calnexin, an ER chaperone protein, interacts with the transmembrane segments containing the acidic amino acids and induces channel degradation through the proteasome pathway [18]. p11, annexin 2 light chain, binds to aa 74–103 in the N-terminus of Na_v_1.8 to promote translocation of the channel to the plasma membrane [8]. Specific knockout of p11 in nociceptive DRG neurons reduces the TTX-R sodium current density and causes a dramatic loss of membrane-associated Na_v_1.8 [21]. As p11 has been shown to mask an ER-localization signal in TASK-1 to promote the surface expression of that channel [22], the role of p11 in promoting Na_v_1.8 trafficking from the ER needs to be evaluated.

Anchoring Na_v_s on the plasma membrane is another key step in the regulation of their surface expression (Fig. 2). Contactin, a cell adhesion molecule, interacts with Na_v_1.2 and Na_v_1.3 [23–25] and is also expressed in DRG neurons [26]. Knockout of contactin causes a reduction in the Na_v_1.8 and Na_v_1.9 currents but not in the TTX-S currents in IB4-positive DRG neurons [26]. PDZ domain containing 2 (PDZD2), a protein containing six PDZ domains, was identified as a Na_v_1.8 interacting protein by a yeast two-hybrid screen [19]. A subsequent study reveals that PDZD2 interacts with the second intracellular loop of Na_v_1.7 and Na_v_1.8 and that knockdown of PDZD2 causes a dramatic decrease in the Na_v_1.8 current [27]. However, knockout of PDZD2 does not cause a change in pain behavior and is accompanied by an increase in p11 [27]. PDZ proteins have been reported to retain and stabilize membrane proteins on the plasma membrane [28–30]. Additional research is required to elucidate the roles of both contactin and PDZD2 in regulating peripheral Na_v_s.

Na_v_ β subunits are cell adhesion molecules and have been reported to regulate the surface expression of Na_v_s including Na_v_1.7, Na_v_1.8 and Na_v_1.9 [11, 31–36]. Coexpression of β1 subunit highly increases the current amplitude of Na_v_1.8 but not Na_v_1.7 in *Xenopus* oocytes [31]. Deficiency of β1 subunit leads to a decrease of persistent TTX-R sodium current accompanying with a reduction of surface and intracellular Na_v_1.9 in mouse small DRG neurons [32]. Loss of β2 subunit results in significant decrease of TTX-S sodium current concomitant with reductions in transcript and protein level of TTX-S Na_v_s, particularly Na_v_1.7 [33]. These phenomena make the notion of trafficking regulation of these Na_v_s by β subunits unsure because the change in the total protein level of channels may cause corresponding altered surface expression of these channels. However, coexpression of β3 subunit with Na_v_1.8 in HEK293 cells or *Xenopus* oocytes induces dramatically increased peak amplitude of sodium current [34, 35], in which the trafficking regulation was supported by significantly enhanced surface expression but not total protein level of Na_v_1.8 in coexpressed HEK293 cells [11]. Both TTX-S and TTX-R resurgent currents in small DRG neurons are enhanced by a peptide-mimetic intracellular domain of the β4 subunit [36]. To date, limited data reveal the molecular basis of β subunits in regulating the trafficking of peripheral Na_v_s. The C-terminus of β3 subunit was examined to mediate the increased surface expression of Na_v_1.8 in coexpressed HEK293 cells and the C-terminal peptide of β4 subunit applied in the patch pipette was detected to enhance resurgent currents of Na_v_1.8 [11, 36]. Since the role of β3 subunit in masking the ER-localization motif of Na_v_1.8, anchoring peripheral Na_v_s on the plasma membrane by β subunits needs further precise experiments to provide evidences.

The effects of post-translational modifications on Na_v_s have been studied [37, 38]. Most studies have focused on the phosphorylation of these channels. Na_v_1.8 is phosphorylated by both protein kinase A (PKA) and protein kinase C, but only PKA-mediated Na_v_1.8 phosphorylation promotes the surface expression of this channel [38, 39]. Inhibition of the PKA-mediated surface expression of Na_v_1.8 by brefeldin A, a drug that blocks secretion upstream of the Golgi complex, reveals increased forward trafficking of this channel [38]; however, it is not clear exactly where in the secretory pathway this regulation occurs.

Recently, ubiquitination of Na_v_1.7 and Na_v_1.8 by the E3 ubiquitin ligase NEDD4-2 has been linked to regulation of the trafficking of these channels [37]. Most Na_v_s, including Na_v_1.6, Na_v_1.7 and Na_v_1.8 but not Na_v_1.9, contain a typical PY motif (PP*X*Y) or variant (LP*X*Y) that interacts with NEDD4-2 and is ubiquitinated [37, 40]. In DRGs, NEDD4-2 is diffusely distributed in small neurons [37, 41]. Overexpression of NEDD4-2 in transfected HEK293 cells dramatically reduces both the amount of Na_v_1.7 in the plasma membrane and the Na_v_1.7 current without changing the abundance or the biophysical properties of this channel, while knockout of NEDD4-2 in Na_v_1.8-positive DRG neurons in SNS-Cre mice causes an increase in Na_v_1.7 current density accompanied by a non-significant change in the abundance of the channel in DRGs [37]. These lines of evidence support a role for NEDD4-2 in the negative regulation of Na_v_1.7 surface expression and indicate that, while channel ubiquitination may impede forward trafficking of Na_v_1.7 or enhance endocytosis (Fig. 2), it does not induce channel degradation or changes in channel properties. Interestingly, knockout of NEDD4-2 causes an increase in Na_v_1.8 current density along with a dramatic increase in the abundance of this channel in DRGs [37], indicating that loss of ubiquitination may reduce degradation of Na_v_1.8. Thus, the same post-translational modification has varying effects on different Na_v_s.

The sumoylation of peripheral Na_v_s has yet been reported in primary sensory neurons. However, the sumoylation deficiency of the collapsin response mediator protein 2 (CRMP2) reduces the surface expression of Na_v_1.7 in HEK293 cells and cultured cortical neurons, and dramatically decreases the peak sodium current density in DRG neurons [42]. Since CRMP2 interacts with tubulin heterodimer and promotes microtubule assembly [43], the role of CRMP2 has been proposed to regulate Na_v_1.7 trafficking.

## Axonal transport and localization of Na_v_s in afferent fibers of primary sensory neurons

The distribution of Na_v_s along axons and at nerve terminals is critical for signal transduction in neurons. Na_v_1.7, Na_v_1.8 and Na_v_1.9 are mainly localized in small DRG neurons, which contribute to unmyelinated C fibers and thinly myelinated Aδ fibers. Transport of Na_v_s via vesicles to the nerve terminal along long-distance axons involves several components, including motor proteins, microtubule tracks and scaffold proteins (Fig. 2). To date, a direct link between microtubule regulation and transport of Na_v_s has not been reported. The kinesin superfamily is composed of microtubule-dependent motors, and kinesin-1 is responsible for anterograde axonal transport. Of the three kinesin-1 isoforms, KIF5A and KIF5B are abundantly expressed in DRG neurons [14]. KIF5B interacts with Na_v_1.8 and Na_v_1.9 but not Na_v_1.6 and Na_v_1.7, while KIF5A weakly interacts with Na_v_1.8 [14]. Overexpression of KIF5B increases the cell-surface and axonal distribution of Na_v_1.8 and simultaneously enhances the Na_v_1.8 current in the soma and axon of cultured DRG neurons, which indicates that, similar to forward trafficking, the anterograde axonal transport of Na_v_1.8 occurs via a mechanism involving motor proteins [14]. Knockdown of KIF5B decreases the current density of Na_v_1.8 in the soma of cultured DRG neurons, indicating a physiological role for KIF5B in promoting channel trafficking [14]. Whether KIF5B promotes the forward trafficking and axonal transport of Na_v_1.9 in primary sensory neurons has yet to be determined.

The scaffold proteins that regulate the axonal transport and localization of Na_v_s are composed of several molecules. Knockout of the cell adhesion protein contactin causes a reduction in the expression of Na_v_1.8 and Na_v_1.9 in unmyelinated fibers of the sciatic nerve [26]. Knockout of the E3 ubiquitin ligase NEDD4-2 dramatically increases the level of Na_v_1.7 in the sciatic nerve [37]. Considering the critical roles that contactin and NEDD4-2 play in the trafficking of Na_v_s, similar mechanisms may underlie axonal transport and localization of these channels. The fact that knockout of NEDD4-2 induces an increase in Na_v_1.8 level in DRGs but not in sciatic nerves [37] provides additional evidence to support the supposition that the NEDD4-2-mediated ubiquitination of Na_v_1.8 only affects the degradation and not the localization of this channel. Recent study showing an interaction between ankyrin G with an ankyrin-binding motif of Na_v_1.8 and a colocalization of Na_v_1.8 with ankyrin G at the nerve terminal of mouse hindpaw skin implies a role of ankyrin G in the axonal localization of Na_v_1.8 [44]. Most importantly, although annexin 2 light chain p11 has been shown to promote Na_v_1.8 translocation to the plasma membrane [8, 21], axonal localization of Na_v_1.8 in sciatic nerve and dorsal root or in cultured DRG neurons has not yet been examined in the p11 knockout mice. p11 together with PDZD2 and β1 subunit are also proposed to act as a lipid raft-sorting factor for Na_v_1.8 in the axons of DRG neurons because they have been shown to be partitioned into lipid rafts [15].

The abundance of Na_v_s, including Na_v_1.7, Na_v_1.8 and Na_v_1.9, is increased in sciatic nerves of animal models with peripheral nerve injury and inflammation [45–49]; however, the molecular mechanisms underlying the axonal transport and localization of these channels in pathological conditions are rarely investigated. In a mouse model with spared nerve ligation, downregulation of NEDD4-2 was thought to be linked to increased Na_v_1.7 level in the sciatic nerve because of a similar phenotype caused by knockout of NEDD4-2 [37]. Recently a potential relationship of increased axonal Na_v_1.8 with KIF5B comes from the result that in peripheral inflammation induced by complete Freund’s adjuvant, increased KIF5 and Na_v_1.8 accumulation were observed in the sciatic nerve. However, the antibody against KIF5 (Catalog Number: MAB1614; Merck Millipore—Chemicon International) was later detected to display low affinity against KIF5B but high affinity against KIF5A and KIF5C as reported by DeBoer et al. [50]. Although KIF5B participates anterograde axonal transport of Na_v_1.8 in the physiological condition [14], the interpretation regarding a potential correlation of the increased axonal transport of Na_v_1.8 with KIF5B in the pathological condition should be revised because of our unpublished result that KIF5B was not increased in the sciatic nerve of rat with peripheral inflammation using the antibody specifically against KIF5B (provided by Drs. Gerardo Morfini and Scott T. Brady). Therefore, the molecular mechanisms underlying the axonal transport and localization of Na_v_1.8 and Na_v_1.9 in pathological conditions remain to be explored.

## Localization of Na_v_1.6 at the nodes of Ranvier in myelinated fibers of primary sensory neurons

The composition of a myelinated fiber in the peripheral nerve system includes the node of Ranvier, paranode, juxtaparanode and internode. The node of Ranvier plays a central role in impulse propagation via salutatory conduction in myelinated fibers. Usually, the channel density at the node is much higher than that in the rest of the nerve fiber [51]. Na_v_1.6 in adult primary sensory neurons in particular, is highly enriched at the nodes of Ranvier [52, 53].

The mechanism of Na_v_ localization at the nodes of Ranvier has been studied for over a decade. The formation of a node of Ranvier is necessary for Na_v_ accumulation. Knockout of a cell adhesion molecule, the 186 kDa isoform of neurofascin (NF-186) that is recruited by Schwann cell-secreted gliomedin, leads to the disruption of nodes and the absence of Na_v_ clusters in sciatic nerves in mice [54]. NF-186 binds the submembrane cytoskeletal protein ankyrin G. Ankyrin G interacts with βVI spectrin and provides scaffolding for the recruitment of a group of functional proteins at the nodes of Ranvier (Fig. 2). Spectrins link the protein complex containing the Na_v_ channel to the actin-based cytoskeleton at the nodes of Ranvier [55, 56]. Recently, the conditional knockout of ankyrin G in DRG neurons or retinal ganglion cells demonstrates that ankyrin G function in Na_v_ clustering can be compensated by ankyrin R [57]. Both ankyrin G-βVI spectrin and ankyrin R-βI spectrin are recruited from a pre-existing pool of unclustered protein complexes to the nodes of Ranvier [57].

For Na_v_1.6 localization in the nodes of Ranvier, an ankyrin G-binding motif (VPIALGESD; corresponding to VPIAVGESD between aa 1094–1102 in murine Na_v_1.6) within the second intracellular loop of rat Na_v_1.2 [58] is sufficient for targeting CD4 chimera proteins to the nodes of Ranvier in rat DRG neuron-Schwann cell myelinating co-cultures [17]. Mutation of the conserved glutamic acid residue at E1100 within the ankyrin G-binding motif blocks Na_v_1.6 targeting to the nodes of Ranvier in neurons of the somatosensory cortex in in utero brain electroporation experiments [17]. Thus, the ankyrin G-binding motif is necessary and sufficient for clustering Na_v_1.6 at the nodes of Ranvier in both peripheral and central nerve systems.

Although knockout of the sodium channel β1 subunit causes a defect in paranodal structure in both sciatic nerves and optic nerves, Na_v_s are still localized to the nodes of Ranvier in sciatic nerves [59]. Na_v_1.6 is also found in the nodes of Ranvier in sciatic nerves following knockout of the sodium channel β2 subunit [60]. Recently, a mutant form of Na_v_1.6, in which casein kinase phosphorylation sites within the second intracellular loop were mutated, was found to efficiently cluster at the nodes of Ranvier, indicating that regulation of casein kinase activity is not essential for node targeting [17].

## Conclusion

Na_v_s determine neuronal excitability and play a vital role in sensory transmission. Thus, Na_v_s, specifically Na_v_1.7 and Na_v_1.8, are key drug targets for pain treatment, with pharmacological companies expending a lot of resources to screen for selective blockers of these channels. The subcellular distribution of Na_v_s is regulated by trafficking (Fig. 2), which sometimes offers a quicker and accurate approach for changing channel function. Understanding the molecular mechanisms that promote excessive Na_v_ trafficking in primary sensory neurons of pathological conditions may lead to the identification of pharmacological targets for pain treatment.

## Summary statement

This review highlights the molecular mechanisms involved in Na_v_ trafficking, focusing on mechanisms that regulate surface expression, axonal distribution and localization to the nodes of Ranvier in adult primary sensory neurons. It also discusses the strategies used to study these mechanisms.

## Abbreviations

Na_v_: voltage-gated sodium channel
DRG: dorsal root ganglion
PCR: polymerase chain reaction
TTX-S: tetrodotoxin-sensitive
TTX-R: tetrodotoxin-resistant
TrkA: tropomyosin receptor kinase A
NF200: 200 kDa neurofilament subunit
IB4: isolectin B4
ER: endoplasmic reticulum
TFR1: transferring receptor 1
PDZD2: PDZ domain containing 2
PKA: protein kinase A
NF-186: 186 kDa isoform of neurofascin
